# Health-related quality of life and social determinants of health following COVID-19 infection in a predominantly Latino population

**DOI:** 10.1186/s41687-022-00473-8

**Published:** 2022-06-23

**Authors:** Kathleen R. Case, Chen-Pin Wang, Meredith G. Hosek, Sarah F. Lill, Alexandra B. Howell, Barbara S. Taylor, James Bridges, Daniel J. MacCarthy, Paula Winkler, Joel Tsevat

**Affiliations:** 1 Center for Research to Advance Community Health (ReACH), Joe R. and Teresa Lozano Long School of Medicine, The University of Texas Health Science Center at San Antonio, San Antonio, TX USA; 2 Division of General and Hospital Medicine, Department of Medicine, Joe R. and Teresa Lozano Long School of Medicine, The University of Texas Health Science Center at San Antonio, San Antonio, TX USA; 3 Department of Population Health Sciences, Joe R. and Teresa Lozano Long School of Medicine, The University of Texas Health Science Center at San Antonio, San Antonio, TX USA; 4 Joe R. and Teresa Long School of Medicine, The University of Texas Health Science Center at San Antonio, San Antonio, TX USA; 5 School of Public Health, The University of Texas Health Science Center at Houston, San Antonio Campus, San Antonio, TX USA; 6 Division of Infectious Diseases, Department of Medicine, Joe R. and Teresa Lozano Long School of Medicine, The University of Texas Health Science Center at San Antonio, San Antonio, TX USA; 7 School of Nursing, University of Texas Health Science Center, San Antonio, TX USA; 8 South Central Area Health Education Center, University of Texas Health Science Center, San Antonio, TX USA

**Keywords:** COVID-19, Quality of life, Latinos, PROMIS-29 + 2, Patient-reported outcomes, Social determinants of health, Long COVID, Post-acute sequelae of COVID-19, Health disparities

## Abstract

**Background:**

As the COVID-19 pandemic evolves, more information is needed on its long-term impacts on health-related quality of life (HRQoL) and social determinants of health (SDoH). The aim of the study was to assess HRQoL and SDoH among a predominantly Latino population of COVID-19 survivors and to compare effects in Latinos versus non-Latinos.

**Methods:**

This cross-sectional study consisted of a survey (in English and Spanish) of COVID-19 survivors from December 2020 to July 2021. The study assessed sociodemographic data, clinical characteristics, and SDoH, consisting of 10 COVID-19—related concerns. The PROMIS-29 + 2 (PROPr) measure, which captures 8 HRQoL domains and a preference-based health utility, was used to assess HRQoL. Bivariate analyses included chi-square tests and t-tests. Generalized linear models were conducted for multivariable analyses.

**Results:**

Of 230 respondents (6.3% response rate), the mean [SD] age was 43.1 [14.3] years; 83.0% were Latino; the mean [SD] time since diagnosis was 8.1 [3.2] months; and 12.6% had a history of hospitalization with COVID-19. HRQoL scores were slightly worse than population norms on all domains, especially anxiety; the mean [SD] PROPr health utility was 0.36 [0.25]. Domain scores were similar by ethnicity except for cognitive function—abilities, where scores were lower in Latinos. Multivariable analyses revealed that: (1) financial concerns were associated with worse health utility, as well as worse scores on all 8 PROMIS domains; (2) interpersonal conflict was associated with worse health utility and worse scores on 6 of the 8 PROMIS domains (anxiety, depression, fatigue, sleep disturbance, social function, and pain interference); and (3) Latino ethnicity was only associated with 1 PROMIS domain (cognitive function—abilities) after controlling for covariates.

**Conclusion:**

COVID-19 infection is associated with HRQoL decrements long after the acute infection, and financial concerns and interpersonal conflict are particularly associated with worse HRQoL.

## Background

The COVID-19 pandemic has claimed more lives in the U.S. than any previous pandemic and has caused substantial morbidity [1, 2]. In addition to the concerns associated with acute COVID-19 infection, research is emerging on the long-term impacts on the physical, mental, and social well-being of survivors [3–22]. While previous research indicates that COVID-19 survivors experience lingering symptoms adversely affecting physical and mental health, cognitive function, energy/fatigue, and sleep, thus far, most of the research has been conducted on small, homogenous samples with short follow-up periods, generally 6 or fewer months between diagnosis and assessment [11, 19, 23–25]. In addition, only a limited number of studies have examined multiple domains of health-related quality of life (HRQoL) in COVID-19 survivors. [14, 20, 21, 25–27]

Notably, the pandemic has also underscored racial and ethnic disparities, as minority populations have been disproportionately impacted [28]. Specifically, Latinos have suffered higher rates of COVID-19 infection, hospitalization, and death as compared with non-Latino whites [29, 30]. As of February 2022, Latinos were 1.5 times as likely to have contracted COVID-19, 2.4 times as likely to be hospitalized, and 1.9 times as likely to die from the disease as non-Latino whites [29]. While disparities in infection and mortality rates among Latinos are well-documented, to date, very little research has examined racial and ethnic differences in the recovery experience of COVID-19 survivors. [26]

The purpose of this study was to investigate the long-term impact of COVID-19 on HRQoL and social determinants of health (SDoH) among a majority Latino population of COVID-19 survivors in Texas, and to compare effects in Latinos versus non-Latinos. We assessed the PROPr health utility score, a preference-based measure of overall health utility constructed from the PROMIS-29 + 2 HRQoL measure. [31]

## Methods

### Study design and participants

This cross-sectional study consisted of a survey of COVID-19 survivors from metropolitan San Antonio, Texas enrolled in the COVID-19 Infectious Diseases Outpatient Clinic (CIVOC) cohort registry, created in March 2020. All individuals in the registry were diagnosed with COVID-19 by a positive PCR test either as an inpatient or outpatient. Study participants were recruited from the CIVOC registry via postal mail; the mailing included a link to the REDCap survey and instructions for obtaining a paper copy of the survey. Multiple modes of survey administration (electronic and paper) were used to increase the response rate; previous research suggests that mode of survey administration does not impact the reliability or validity of PROMIS scales [32]. Inclusion criteria included (1) a history of COVID-19 infection diagnosed from March 13, 2020–November 21, 2020; (2) age 18 years or older with validated age, sex, and ethnicity data; (3) reading and writing fluency in English or Spanish; (4) residence in Bexar County, Texas; and (5) complete and deliverable mail address information. Data were collected from December 2020 to July 2021; participants participated in the survey (English or Spanish) online through REDCap or returned a paper survey. Two mailings were used to recruit participants for the study; the first occurred in December 2020, the second mailing was sent in April 2021 to those who did not respond to the first study invitation. Consent was obtained electronically via REDCap or on paper if participants chose to return the survey by mail. Participants were compensated $20 following completion of the survey. This study was approved by the University of Texas Health Science Center at San Antonio Institutional Review Board (HSC-2020-0765E).

### Data sources and variables

#### HRQoL and SDoH

To assess HRQoL, we used the 31-item PROMIS-29 + 2 (PROPr) measure, which addresses 8 health domains: anxiety, depression, fatigue, pain interference, physical function, sleep disturbance, ability to participate in social roles and activities, and cognitive function—abilities. Cognitive function—abilities assesses items related to cognitive functioning including memory and concentration [33]. The PROMIS measures have demonstrated reliability and construct validity in diverse populations [34]. PROMIS scoring includes calculation of a T-score, which is used to compare sample scores versus the general population (mean = 50, SD = 10). Higher T-scores correspond to higher levels of the construct (for example, greater anxiety, better physical function). In addition, the measure produces a PROPr score, an overall health utility ranging from -0.022 (worst) to 1.0 (best). [35]

To assess SDoH, we developed a set of items addressing COVID-19 concerns and needs. The question stem was, “Based on your experience with COVID-19, which of the following are you most worried about over the next several months? (Please select ALL that apply),” and the response options were: (1) ongoing COVID-related health impacts; (2) being able to access COVID-related healthcare, including vaccines or treatments; (3) having enough money to cover essential expenses (food, housing, bills, etc.); (4) getting or keeping a job; (5) education/schooling for self or others; (6) childcare; (7) caring for sick/aged family members; (8) having a stable place to live; (9) conflict within the home or with family members; and (10) other. Variables were created for each of the potential response options; coding corresponded for 1 “selected” and 0 “not selected”.

#### Demographics and clinical variables

We obtained clinical data for the date of COVID-19 diagnosis and hospitalization records from the CIVOC registry. We defined history of hospitalization as either hospitalization for COVID-19 infection, or a COVID-19 diagnosis made during hospitalization for another primary diagnosis. For demographic characteristics, the CIVOC student research team verified race, ethnicity, and date of birth for all patients in the COVID-19 cohort by searching for their medical record number (MRN) in the Electronic Health Record to confirm the integrity of the data transfer into the CIVOC registry. Data on respondents’ age, sex, ethnicity (Latino/ non-Latino) were also cross validated between the survey and registry.

### Statistical analysis

The analytic dataset consisted of respondents with complete PROMIS-29 + 2 data. Eighty-four participants with incomplete PROMIS-29 + 2 data were removed from the analyses and 2 additional participants with missing covariates were also excluded from the analyses. In addition to calculating descriptive statistics, we conducted bivariate and multivariable analyses. Bivariate analyses included chi-square tests for categorical variables and t-tests for continuous measures. The effect size of ethnicity on each HRQoL and COVID-19 concern variable was calculated as the difference of means between ethnic groups divided by the pooled standard deviation (Cohen’s d formula). Multivariable analyses consisted of 9 multivariable regression models, in which the outcome variables were the PROPr health utility score and the 8 PROMIS domains. Exposure variables in the multivariable analyses included Latino ethnicity, age, sex, prior hospitalization for COVID-19, months since diagnosis, and SDoH. The stepwise forward selection and backward elimination method was used to select variables in the final models, where *p* < 0.15 was used as the entry significance level and *p* < 0.05 as the retention significance level. Because Latino ethnicity was an exposure of interest, it was included in the final models regardless of statistical significance.

## Results

**Fig. 1:**
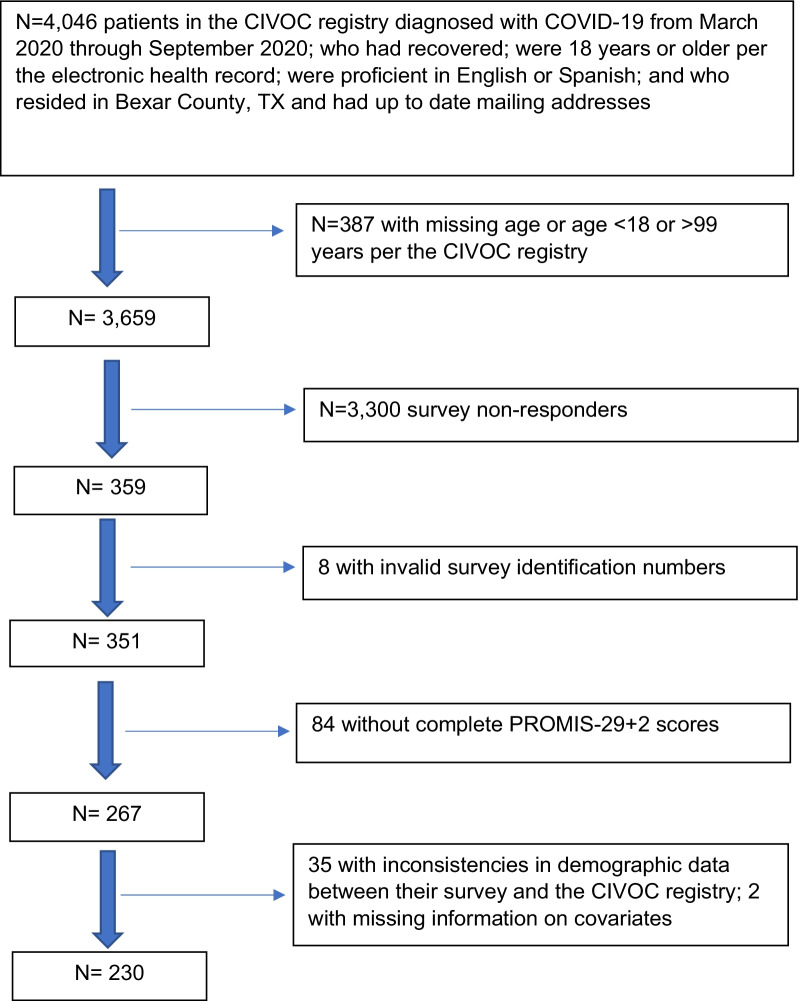
Study flow diagram

The total number of respondents to the survey was 359, of whom we excluded 129, for a final sample of 230 (Fig. 1). The response rate was 6.3%; corresponding to those who completed all variables of interests (n = 230), divided by those eligible to complete the study (n = 3659). Compared with eligible participants in the CIVOC registry who did not complete our survey, our sample was slightly over-represented by women and participants with outpatient (vs. inpatient) COVID-19 diagnoses (Table 1). Overall, the mean [SD] age 43.1 [14.3] years, 83.0% of COVID-19 survivors were Latino, 68.7% were female, and 12.6% had been hospitalized. The mean [SD] number of months since diagnosis was 8.1 [3.2] (Table 2). Demographic results were similar for Latinos vs. non-Latinos except that the time elapsed since COVID-19 diagnosis was greater for Latinos.

**Table 1:** Demographics of the included sample versus non-responders

	Included sample (n = 230)	Non-responders (N = 3429)*	*p* value
Latino ethnicity, n (%)	191 (83.0)	2459 (78.8)	0.13
Mean (SD) age, years	43.1 (14.3)	44.9 (15.4)	0.10
Male sex, n (%)	72 (31.3)	1477 (43.5)	< 0.001
Prior hospitalization with COVID-19, n (%)	29 (12.6)	587 (17.1)	< 0.001

*Among 4046 unique patients in the registry, 387 were excluded for ages < 18 or > 100 or missing age

**Table 2:** Comparison of demographic and clinical characteristics by ethnicity

	Overall sample (n = 230)	Latino/as (n = 191)	Non-Latino/as (n = 39)	*p* value
Age, M (SD)	43.1 (14.3)	42.7 (14.4)	44.7 (14.1)	0.45
Male sex, n (%)	72 (31.3)	61 (31.9)	11 (28.2)	0.65
Prior hospitalization for COVID-19, n (%)	29 (12.6)	22 (11.5)	7 (17.9)	0.27
Months since diagnosis, M (SD)	8.1 (3.2)	8.3 (3.2)	7.0 (3.2)	0.02

HRQoL scores were slightly worse than population norms for 7 of the 8 PROMIS domains, and the mean [SD] PROPr health utility was 0.36 [0.25] (Table 3). In bivariate analyses comparing HRQoL scores by ethnicity, cognitive function—abilities scores were lower among Latinos than non-Latinos. In addition to being statistically significant, the differences between cognitive function—abilities scores also met the threshold for minimal clinically important difference of 3 T-scores points [36]; the effect size for the difference was small. PROPr health utility scores did not differ for Latinos as compared with non-Latinos. Among SDoH, the most prevalent were health-related impacts (67.4%), as well as financial (45.2%), and employment (31.7%) concerns. Significantly greater proportions of Latinos as compared with non-Latinos reported concerns regarding access to healthcare (24.6% vs. 10.3%; *p* = 0.05) and finances (49.2% vs. 25.6%; *p* = 0.007). Among the SDoH variables that were statistically different between Latinos and non-Latinos, financial concerns had the largest effect size (0.36), followed by access to health care (0.27); both effect sizes were small.

**Table 3:** Health-related quality of life and COVID-19 concerns

	Overall sample (n = 230)	Latino/as (n = 191)	Non-Latino/as (n = 39)	Effect size	*p* value
*PROMIS scores, mean (SD)*					
PROPr utility*	0.36 (0.25)	0.35 (0.25)	0.41 (0.27)	0.16	0.15
Physical function^†^	46.3 (9.8)	46.2 (9.8)	46.9 (10.3)	0.05	0.71
Anxiety	56.7 (11.2)	57.3 (11.1)	54.0 (11.1)	0.21	0.08
Depression	53.1 (10.6)	53.4 (10.6)	51.4 (10.7)	0.13	0.30
Fatigue	53.4 (12.1)	53.2 (12.1)	54.1 (11.9)	0.05	0.69
Sleep disturbance	54.1 (8.8)	54.5 (8.7)	51.9 (9.1)	0.21	0.09
Social function	50.2 (10.5)	50.2 (10.4)	49.9 (11.1)	0.02	0.83
Pain interference	53.1 (10.9)	53.2 (10.7)	52.6 (11.5)	0.04	0.65
Cognitive function—abilities	46.7 (9.2)	45.9 (9.0)	50.5 (9.0)	0.36	0.003
*COVID-19 concerns*^‡^*, n (%)*					
Health-related impacts	155 (67.4)	131 (68.6)	24 (61.5)	0.10	0.39
Access to healthcare	51 (22.2)	47 (24.6)	4 (10.3)	0.27	0.05
Financial	104 (45.2)	94 (49.2)	10 (25.6)	0.36	0.007
Employment	73 (31.7)	65 (34.0)	8 (20.5)	0.22	0.10
Education	53 (23.0)	47 (24.6)	6 (15.4)	0.16	0.21
Childcare	20 (8.7)	16 (8.4)	4 (10.3)	0.05	0.70
Caring for family members	52 (22.6)	39 (20.4)	13 (33.3)	0.21	0.08
Housing	25 (10.9)	22 (11.5)	3 (7.7)	0.09	0.48
Interpersonal conflict	31 (13.5)	28 (14.7)	3 (7.7)	0.16	0.25
Other	15 (6.5)	12 (6.3)	3 (7.7)	0.04	0.74

*Values can range from − 0.022 (worst) to 1.0 (best)

^†^T-scores (population norm = 50, SD = 10; higher scores indicate higher levels of the particular domain, e.g., better physical function, more anxiety)

^‡^Proportion corresponds to percentage of participants who selected each response; more than one response was allowed

In multivariable regression analyses of HRQoL outcomes, the SDoH most associated with HRQoL scores were financial concerns and interpersonal conflict (Table 4). Financial concerns were significantly associated with worse health utility and all 8 of the PROMIS domains. Interpersonal concerns were associated with worse health utility and social function; greater anxiety, depression, fatigue, pain interference, and sleep disturbance. In addition to financial and interpersonal concerns, health-related impacts of COVID-19 were also significantly associated with greater anxiety; concern over education for themselves or others was associated with reduced pain interference. For demographic and clinical covariates, the results differed across models. Older age was significantly associated with worse physical function, pain interference, and social function. Longer time since diagnosis and Latino ethnicity were significantly associated with worse cognitive function—abilities.

**Table 4:** Multivariable regression analyses examining the associations between demographics, clinical characteristics, COVID-19 concerns and health-related quality of life domains

Variable	B estimate (SE)	*p* value
*Health utility*		
Latino ethnicity	− 0.03 (0.04)	0.55
COVID-19 concerns		
Financial	− **0.11 (**− **0.03)**	< 0.001
Interpersonal conflict	− **0.15 (0.05)**	0.001
Adjusted R^2^ = 0.10		
*Physical function*		
Latino ethnicity	− 0.14 (1.64)	0.93
Age	− **0.21 (0.04)**	< 0.001
COVID-19 concerns		
Financial	− **4.39 (1.24)**	< 0.001
Adjusted R^2^ = 0.12		
*Anxiety*		
Latino ethnicity	1.32 (1.81)	0.47
COVID-19 concerns		
Health-related impacts	**3.92 (1.44)**	0.007
Financial	**4.52 (1.39)**	0.006
Interpersonal conflict	**9.03 (2.02)**	< 0.001
Adjusted R^2^ = 0.18		
*Depression*		
Latino ethnicity	0.56 (1.81)	0.76
COVID-19 concerns		
Financial	**4.10 (1.39)**	0.004
Interpersonal conflict	**6.80 (2.00)**	< 0.001
Adjusted R^2^ = 0.09		
*Fatigue*		
Latino ethnicity	− 2.69 (2.04)	0.30
COVID-19 concerns		
Financial	**5.22 (1.57)**	0.001
Interpersonal conflict	**7.96 (2.26)**	<.001
Adjusted R^2^ = 0.13		
*Sleep disturbance*		
Latino ethnicity	1.35 (1.49)	0.36
COVID-19 concerns		
Financial	**3.45 (1.15)**	0.003
Interpersonal conflict	**6.08 (1.65)**	< 0.001
Adjusted R^2^ = 0.11		
*Social function*		
Latino ethnicity	1.40 (1.74)	0.42
Age	− **0.19 (0.05)**	< 0.001
COVID-19 concerns		
Financial	− **3.72 (1.34)**	0.006
Interpersonal conflict	− **7.66 (1.93)**	< 0.001
Adjusted R^2^ = 0.14		
*Pain interference*		
Latino ethnicity	0.02 (1.82)	0.99
Age	**0.20 (.05)**	< 0.001
COVID-19 concerns		
Financial	**4.30 (1.42)**	0.003
Education	− **3.37 (1.69)**	0.05
Interpersonal conflict	**4.93 (2.04)**	0.02
Adjusted R^2^ = 0.14		
*Cognitive function—abilities*		
Latino ethnicity	− **3.34 (1.59)**	0.04
Months since diagnosis	− **0.58 (0.18)**	0.002
COVID-19 concerns		
Financial	− **2.80 (1.20)**	0.02
Adjusted R^2^ = 0.08		

Significant findings are reported (*p* < .05; bold font), as well as the association between Latino ethnicity and each outcome

## Discussion

In this relatively large, predominantly Latino, cross-sectional study of COVID-19 survivors, we assessed SDoH and HRQoL on average 8 months post-diagnosis. Notable findings include slightly worse HRQoL scores relative to population norms in 7 of the 8 PROMIS domains, as well as reduced health utility.

To date, there has been limited research examining HRQoL among COVID-19 survivors beyond a short time after diagnosis and among ethnically diverse samples. In 1 of the largest studies of COVID-19 survivors (n = 819), Ganesh and colleagues assessed the impact of COVID-19 infection on HRQoL of COVID-19 survivors drawn from the Mayo Clinic [21]. Overall, respondents reported significantly worse scores (greater than 1 SD) as compared with the expected population mean with respect to social roles, pain, fatigue, physical function, and sleep. Their follow-up time between COVID-19 diagnosis and survey completion was only 68 days, however. In addition, the sample was primarily non-Latino white (86%) and additional HRQoL constructs such as depression, anxiety, and cognitive function were not assessed. In a study with a longer follow-up between diagnosis and survey administration (approximately 6 months), researchers found that, among COVID-19 survivors (n = 177), nearly a third reported at least 1 persistent physical symptom [22]. In a recent study of survivors of severe COVID-19 (i.e. requiring intensive care unit admission), researchers found at 6 months follow up that respondents (n = 132) did not differ from the U.S. population on PROMIS 29-2 domain scores, with the exception of physical function (mean [SD] 44.2 [11.0]) and global mental health scores (mean [SD] 43.1 [10.8]) [27]. Importantly, that study did not examine differences between Latinos and non-Latinos and did not examine the relationships between SDoH and HRQoL outcomes.

In our study, the HRQoL domain that was most abnormal relative to population norms was anxiety. In multivariable analysis, 3 SDoH factors were associated with greater anxiety: health-related impacts, financial concerns, and interpersonal conflict within the home or with other family members. These results are consistent with a systematic review demonstrating that anxiety is 1 of the most common symptoms of PASC [24]. In that review, across 7 studies that reported symptoms of anxiety among COVID-19 survivors, 30% of subjects were diagnosed with generalized anxiety disorder. In a study that utilized PROMIS to assess anxiety among hospitalized patients with COVID-19 in Israel (n = 90), patients had moderately high levels of anxiety (mean [SD] T-score, 57.7 [11.9]). [5]

Furthermore, the consistent associations between 2 SDoH, financial concerns and interpersonal conflict, with health utility and the PROMIS domains demonstrate the salience of these SDoH as it relates to HRQoL. Recent research by Hanmer has also shown that SDoH are associated with health utility. Specifically, Hanmer found that health utility scores were associated with education, income, employment, difficulty getting to medical appointments, food and financial insecurity, intimate partner violence, stress, and social situation [31]. Importantly. economic impacts may be particularly salient for our study’s majority Latino population, since Latino workers suffer higher unemployment rates than non-Latino whites [37]. In one of the only studies that has investigated the impacts of COVID-19 on mental health outcomes among Latinos, researchers found that economic strain (e.g. job loss, inability to afford rent) due to COVID-19 was associated with increased anxiety symptoms, while psychosocial consequences (e.g. loss of important relationships, substance use) were associated with both increased anxiety and depressive symptoms among a sample of young adults [38]. Our findings differ from that study in that financial concerns were associated with both increased anxiety and depression. Notably, the study by Villatoro and colleagues included Latino young adults and was not specific to COVID-19 survivors. Financial concerns may be more prominent for COVID-19 survivors as many have to take significant amounts of time off work while they recover from the illness [39]. While previous research has demonstrated associations between SDoH, such as COVID-19-related financial concerns, with negative mental health outcomes [38, 40, 41]; no studies have examined their impact on physical health PROMIS domains, such as physical function, pain interference, and fatigue.

Finally, specific to cognitive function—abilities, financial concerns, along with Latino ethnicity and time since diagnosis, were associated with worse scores. In other words, the longer since diagnosis, the worse the cognitive function. Here, our study is limited by the cross-sectional design, so we do not know whether cognitive function changed over time. The lower cognitive function—abilities scores among Latinos could be explained by variables that we did not measure. Many previous studies have demonstrated that diminished cognitive function, or “brain fog,” is common in PASC [24, 26, 33]. In an analysis of factors contributing to diminished cognitive function due to COVID-19 infection, Baker and co-authors attributed: (1) pre-existing conditions such as advanced age, diabetes, or obesity; (2) COVID-19 inflammation leading to hypoxia, ischemia, and neuronal injury; and (3) treatment effects such as prolonged sedation [42]. While we did not collect information on comorbidities, research suggests that Latinos may suffer more adverse outcomes from COVID-19 due to greater rates of pre-existing conditions, such as hypertension, diabetes, and obesity [43, 44]. In South Texas, diabetes and obesity are more common among Latinos than non-Latinos [45]. These same underlying conditions may also negatively impact cognitive function among COVID-19 survivors. Moreover, our findings regarding cognitive function should be interpreted with the consideration that although the survey was administered in both English and Spanish, we did not assess additional acculturation measures including primary language spoken at home, which may impact scores on the cognitive function—abilities measure. As a result, reduced cognitive function may be confounded by unmeasured factors as opposed to reflecting true differences in function.

Our study provides important insight into HRQoL within a predominantly Latino population on average 8 months following COVID-19 diagnosis. As noted previously, most research on COVID-19 survivors has included mostly non-Latino white samples, with 6 months or less time from diagnosis to follow-up [24]. In a systematic review by Groff and colleagues of 57 articles examining short and long-term consequences of COVID-19, only 13 had larger sample sizes than our study [24]. Of those 13 studies, only 3 were conducted in the U.S. [4, 12, 46] and only 1 had a follow-up of 6 months or longer [47]. Similarly, the more recent study by Neville and co-authors had a smaller sample size (n = 132) than our study, and surveyed participants at 6 months post-hospital discharge. [27]

While the current study has numerous strengths, a few limitations warrant discussion. First, the study was cross-sectional; therefore, we do not have HRQoL or SDoH data prior to COVID-19 infection, and as such, we do not know if COVID-19 is the direct cause of the reduced HRQoL among survivors. As stated previously, we did not assess acculturation measures, such as language spoken at home, or information regarding comorbidities, which might bias the results in favor of finding a stronger association between Latino ethnicity and reduced cognitive function. Additionally, COVID-19 survivors who participated in our study differed in certain respects from those in the COVID-19 registry as a whole; our sample had greater proportions of women and outpatient COVID-19 diagnoses than non-responders. Therefore, our results are not generalizable to the full COVID-19 registry. Importantly, however, because a larger proportion of non-responders were hospitalized when they were diagnosed with COVID-19, their patient-reported outcomes may be worse than those observed in our sample. Furthermore, the small number of non-Latino participants (n = 39) might have prevented the detection of important significant differences between Latinos and non-Latinos on SDoH and HRQoL variables. An additional limitation is that the measure used to assess SDoH asked which concerns from a list the patient is most worried about, and then instructed participants to select all that apply. Given this wording, some participants may have only selected one concern (the most concerning one), thus partly obscuring the relationships we found between SDoH and HRQoL outcomes.

Another limitation of the study is the low response rate; there are several reasons that may explain this phenomenon. First, our sample was recruited from a registry that was created for clinical care, as opposed to for research. Low response rate to the survey may also be attributed to reluctance to engage in COVID-19 research due to social, political, or religious factors. Finally, an additional limitation is the lack of information regarding patients’ past medical histories, pre-existing conditions, reasons for hospitalization, and the severity of COVID-19 infection. Notably, the registry was created as a tool to allow providers to identify patients who have had COVID-19 infection and arrange appropriate follow up.

In spite of these limitations, our study has many strengths, including assessment of HRQoL at an average of 8 months post-infection, which provides needed insight into the long-term recovery from COVID-19. Furthermore, unlike previous research, our study population is majority minority, with 83.0% of participants identifying as Latino. Our participants were also not limited to those with severe COVID-19 infection requiring hospitalization, as only 12.6% of our sample were diagnosed while hospitalized with COVID-19.

## Conclusions

This study is one of the first to investigate the long-term impact of COVID-19 infection and SDoH among a majority minority patient population. Our results demonstrate that COVID-19 infection can be associated with HRQoL decrements long after the acute infection, and various demographic factors and SDoH are associated with worse HRQoL. Future studies should follow patients longitudinally well beyond the time of infection and should test both medical and social interventions to mitigate HRQoL loss.

## Data Availability

The datasets used and/or analysed during the current study are available from the corresponding author on reasonable request.
